# CT-based screening of sarcopenia and its role in cachexia syndrome in pancreatic cancer

**DOI:** 10.1371/journal.pone.0291185

**Published:** 2024-01-25

**Authors:** Ekaterina Khristenko, Valentin Sinitsyn, Tatiana Rieden, Parker Girod, Hans-Ulrich Kauczor, Philipp Mayer, Miriam Klauss, Vladimir Lyadov

**Affiliations:** 1 Department of Diagnostical and Interventional Radiology, Heidelberg University Hospital, Heidelberg, Germany; 2 Department of Radiology at Medical Educational and Scientific Center University Hospital, Lomonosov Moscow State University, Moscow, Russia; 3 Institute for Diagnostical and Interventional Radiology, Ludwigshafen Clinical Hospital, Ludwigshafen am Rhein, Germany; 4 University College Dublin School of Chemistry, Dublin, Ireland; 5 Moscow City Clinical Cancer Hospital No. 1, Oncology No. 4, Moscow, Russia; Affiliated Hospital of Nanjing University of Chinese Medicine: Jiangsu Province Academy of Traditional Chinese Medicine, CHINA

## Abstract

Since computed tomography (CT) is a part of standard diagnostic protocol in pancreatic ductal adenocarcinoma (PDAC), we have evaluated the value of CT for sarcopenia screening in patients with PDAC, intending to expand the diagnostic value of tomographic studies. In our study, we included 177 patients with available CT images. Two groups were formed: Group 1 consisted of 117 patients with PDAC in various locations and stages and Group 2, or the control group, consisted of 60 "nominally healthy" patients with other somatic non-oncological diseases. The body mass index (BMI) was defined as a ratio of patient’s weight to the square of their height (kg/m^2^). CT-based body composition analysis was performed using commercially available software with evaluation of sarcopenia using skeletal muscle index (SMI, cm^2^/m^2^). Based on the SMI values, sarcopenia was found in 67.5% of patients (79 out of 117) in the first patient group. It was found more frequently in males (42 out of 56; 75%) than in females (37 out of 61; 60.6%). Additionally, we observed a decrease in muscle mass (hidden sarcopenia) in 79.7% in patients with a normal BMI. Even in overweight patients, sarcopenia was found in 50% (sarcopenic obesity). In patients with reduced BMI sarcopenia was found in all cases (100%). Statistically significant difference of SMI between two groups was revealed for both sexes (p = 0,0001), with no significant difference between groups in BMI. BMI is an inaccurate value for the assessment of body composition as it does not reflect in the details the human body structure. As SMI may correlate with the prognosis, decreased muscle mass- especially "hidden" sarcopenia or sarcopenic obesity- should be reported. The use of CT-based evaluation of sarcopenia and sarcopenic obesity will allow for a better treatment response assessment in patients with cancer cachexia.

## Introduction

Cachexia syndrome is a common condition in various pathological entities including, cancer, sepsis, chronic heart disease, and chronic obstructive pulmonary disease (COPD) [1]. Most definitions of cachexia in previous studies commonly described it as a multidimensional syndrome or condition, which include such contributing factors as anorexia, weight loss, chemosensory distortion, inflammation, hypermetabolism, and early satiety. Cachexia led to such outcomes as anaemia, asthenia, psychosocial distress, dyspnoea, dependency upon third persons, toxicity of chemotherapy, and death [2]. Recently, the number of publications in metabolic imaging in general, and cancer cachexia in particular, increased dramatically. Many of them define muscle depletion, or sarcopenia, as a key factor in the development of cancer cachexia syndrome, thus changing the definition of cachexia.

In the literature, cancer cachexia is described as a multifactor syndrome, which is characterized by an ongoing loss of skeletal muscle mass which can take place with or without simultaneous loss of fat mass. Patients with cancer cachexia syndrome don’t fully respond to conventional nutritional support, which leads to progressive functional impairment. In terms of pathophysiology, this syndrome is defined by a negative protein and energy balance which is caused by a combination of abnormal metabolism and reduced food intake [3]. The universally accepted diagnostic criteria for cachexia is weight loss of >5% in the last 6 months or weight loss of > 2% in patients already showing depletion according to current bodyweight and height (body-mass index <20 kg/m) or skeletal muscle mass.

Tumors and inflammatory diseases of the pancreas are often accompanied by the development of severe metabolic disorders associated with weight loss, changes in body structure, and, in particular, a decrease in muscle mass (sarcopenia). The revised European Working Group on Sarcopenia in Older People (EWGSOP2) definition of sarcopenia suggests using muscle strength as the primary diagnostic criterium [4–7]. However, the vast majority of studies showing prognostic value of sarcopenia in cancer used CT-based calculations of muscle mass with specific cut-offs [8]. Despite multiple studies in various cancer type show the importance of CT-based sarcopenia screening [8], the assessment of body composition in pancreatic cancer patients is still far from being routine which is the underlying reason for our study.

Many studies show that in clinical practice, the analysis of body composition allows estimation of isolated loss of muscle mass (sarcopenia) in cancer patients, thereby suggesting the presence of cancer cachexia and assessing the rate of its progression. Changes in the physical condition of the patients may signal risks of performing invasive procedures and predetermine the overall prognosis [9–11]. Today, the diagnostic imaging studies, such as multi-detector computed tomography (MDCT) and magnetic resonance imaging (MRI), are widely used to estimate the body composition and muscle area and mass [12].

Since computed tomography (CT) is a part of standard diagnostic protocol in pancreatic ductal adenocarcinoma (PDAC), we have evaluated the value of CT for sarcopenia screening in patients with PDAC, intending to expand the diagnostic value of tomographic studies. Because low reproducibility may be a result of a poor scan level selection, a clear anatomical reference point (skeletal marker, not the soft tissue or organs) and a standardized approach to calculating the "skeletal-muscular index of L3" is important for analyzing body composition [13].

## Methods

### Ethics approval and consent

Our study was performed according to the Declaration of Helsinki. The local ethical review board of Russian Medical Academy of Continuing Medical Education (RMANPO) issued an ethical approval for the study without further restrictions (from 21^st^ of October 2014, N8). A written informed consent was obtained from the study participants. All relevant data are within the manuscript and its Supporting Information files.

### Patient selection and data collection

We included 177 patients in our study (91 males and 86 females), who underwent diagnostical studies and therapy from March 2009 till April 2014. Group 1 included 117 patients with histologically proven pancreatic cancer (PC) (mean age 64,8±10,5; females– 48%, males 52%). There were 54 (46%) patients with stages I-II (preoperative evaluation) and 63 unresectable stage III-IV (54%) patients. Group 2 included 60 patients who got a CT of the abdomen because of a clinical suspicion of pancreatic disorder but no proven cancer of the pancreas (mainly chronic pancreatitis). There was no statistically significant difference with Group 1 parameters including age and sex distribution. The patients with resectable PC undergone the surgical treatment und those with unresectable stage–chemotherapy.

The body mass index (BMI) was defined as a ratio of patient’s weight to the square of their height (kg/m^2^). The reference values were the generally accepted values of 18.6 kg/m^2^-24.9 kg/m^2^, without sex differentiation.

### Radiological methods and assessment

In this study, we evaluated unenhanced CT-scans with 1.5 mm slice thickness as they provide sufficient diagnostic information for assessment of body composition. CT examinations were carried out using Somatom Sensation MDCT-scanner (64 rows, Siemens) and Discovery 750 dual energy CT-scanner (GE Healthcare). All studies included unenhanced phase, which was used for the analysis. The technical parameters of the studies were the following: 120 kW tube voltage and 170 mA tube current. During the study, the patients were instructed to hold their breath for 9–12 seconds. The range of radiation dose was from 4 to 25 mSv (cumulative dose). For postprocessing, we used the Slice-O-Matic body composition analysis software by TomoVision (Montreal, Canada).

Evaluation of muscle mass (sarcopenia estimation) was performed by one radiologist (EK, 10 years of experience in abdominal imaging) in all the cases. The mean time for the analysis of each patient for the manual assessment was also evaluated.

For the differentiated estimation of the body structure, the muscle tissue area (cm^2^) was determined by two consecutive axial slices performed at the body level of the third lumbar vertebrae. We selected all the striated muscles which included m. psoas major, m. quadratus lumborum, m. erector spinae, m. obliquus internus abdominis, m. obliquus externus abdominis, m. rectus abdominis, and m. transversus abdominis on each of the two following axial CT slices. Then, the sum of the muscle areas for each slice was calculated automatically, followed by the calculation of the arithmetic mean. The ratio of the obtained skeletal muscle area at the L3 level to the patient’s height squared was the "skeletal-muscular index of L3" (SMI, cm^2^/m^2^). The cut-off values of SMI for sarcopenia were accepted according to the recommendations of the leading research team in this field by V. Baracos [1, 12, 14–18]. SMI values, equal or less than 52.4 cm^2^/m^2^ for males and 38.5 cm^2^/m^2^ for females, correspond to CT-based sarcopenia defined as a state in which the percentage of muscle mass is two or more standard deviations lower than its average values in healthy adults of the same age and sex [3, 19, 20]. Fat tissue area was also calculated- separate for the visceral fat area (VFA) and for the subcutaneous fat area (SFA).

### Statistical analysis

Data management was carried out by SAS software release 9.4 (SAS Institute, Cary, North Carolina, USA) and statistical analysis was made using IMB SPSS software, version 24 (IBM Corp.). Quantitative variables presented as medians. Mann–Whitney U test was performed to compare continuous parameters between groups. For categorial parameters, absolute numbers are shown. Two-sided p-values were computed, the differences were considered statistically significant at a P-value of 0.05 or less.

## Results

**Fig 1:**
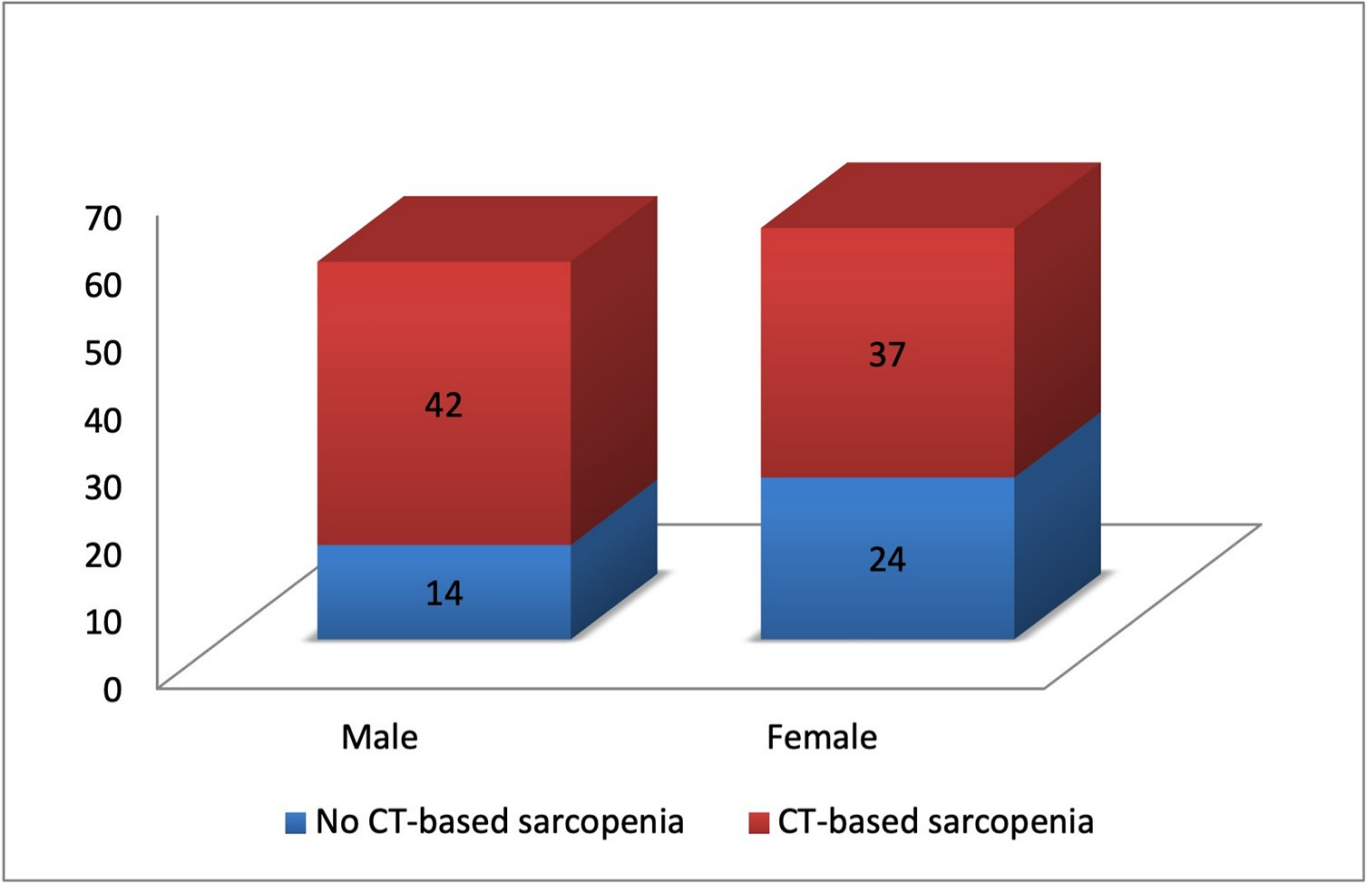
Prevalence of sarcopenia in patients with pancreatic ductal adenocarcinoma (Group 1) classified by gender.

Based on the SMI values, CT-based sarcopenia was found in 67.5% (79 out of 117) of the examined patients in the first patient group with pancreatic ductal adenocarcinoma. In the same patient group, CT-based sarcopenia was more frequently found in males (42 out of 56; 75%) than in females (37 out of 61; 60.6%) although, in both groups sarcopenic patients prevailed (**Fig 1**). We compared the SMI values with the BMI values as the detection of decreased muscle mass in normal weight and overweight patients, so called “hidden sarcopenia”, is of great clinical importance. The results of this comparison are shown in **Table 1**.

**Table 1:** Results of CT body composition analysis in patients with pancreatic cancer (n = 117).

Body composition	All patients (117)	Patients with sarcopenia (79)	Patients without sarcopenia (38)
	Body mass index (kg/m^2^)		
	24.71±4.59	23.84±3.8	26.58±3.79
L3-index men (cm^2^/m^2^) L3-index women (cm^2^/m^2^) Visceral fat area men (cm^2^) Visceral fat women (сm^2^) Subcutaneous fat area men (сm^2^) Subcutaneous fat area women (сm^2^) Total fat area men (сm^2^) Total fat area women (сm^2^)	46.8±7.8 37.38±5.86 156.22±104 108±69.51 120.69±78.6 159.02 ±113.23 255.22±174.32 209.16±211.51	43.71±5.9 33.86±3.37 141.52±90.94 103.69±74.10 108.96±66.49 150.87±126.03 227.41±150.83 191.62±223.10	56.45±4 42.83±4.59 199.95±126.33 115.13±62.62 159.0±98.17 174.35±83.73 347.59±212.48 242.23±190.51

**Fig 2:**
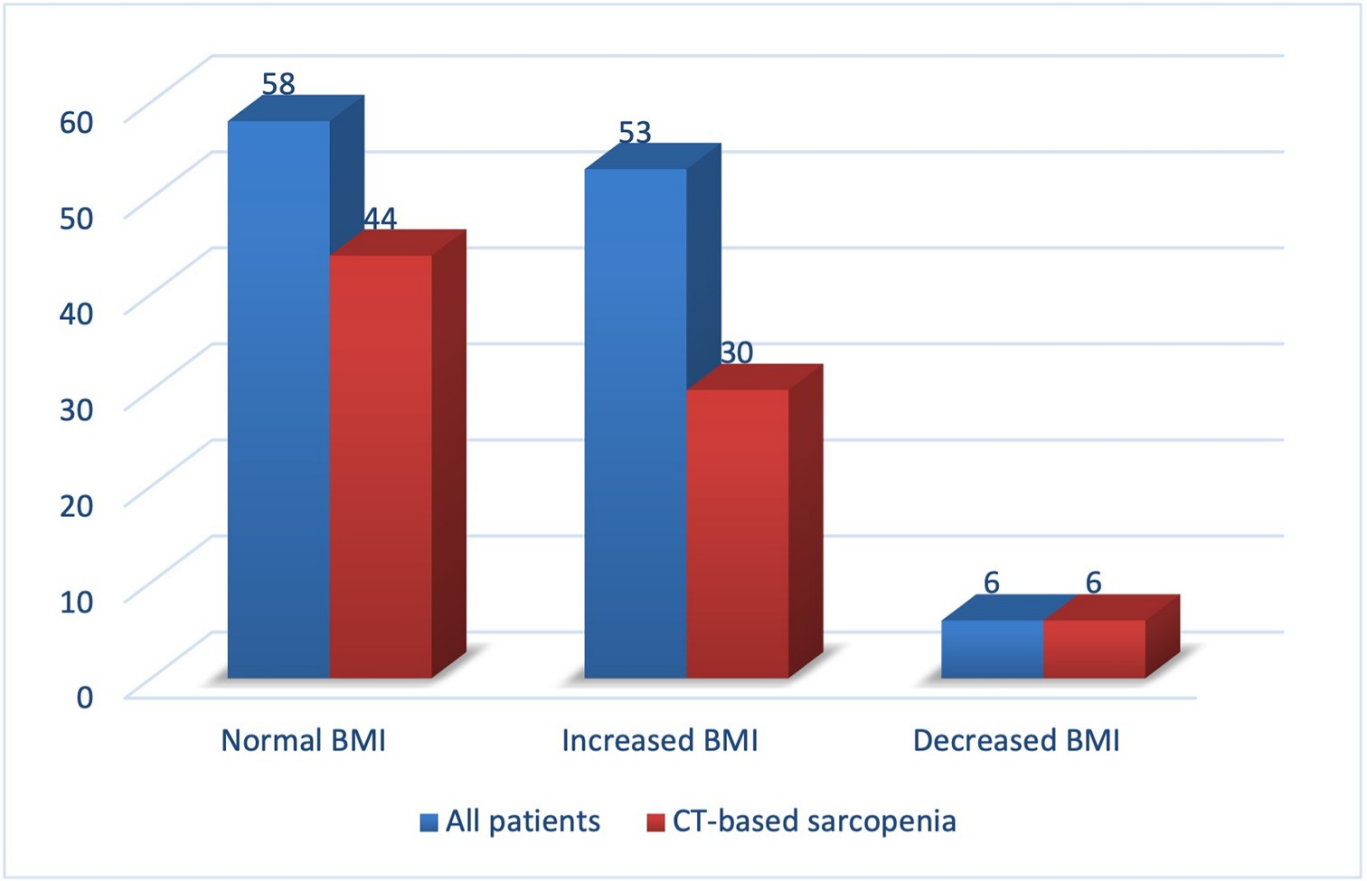
The proportion of sarcopenia in patients with pancreatic ductal adenocarcinoma (Group 1) with different BMI.

**Table 2** and **Fig 2** show the ratios between BMI and SMI in patients with PDAC (Group 1). In patients with normal BMI, we observed a decrease in muscle mass (hidden sarcopenia) in 79.7% of cases. Even in overweight patients (increased BMI), CT-based sarcopenia was found in 50% of cases. Reduced BMI was found in 6 patients with PC and CT-based sarcopenia was found in all cases (100%). It is noteworthy that CT-based sarcopenia was found more frequently in patients with cancer as the BMI value decreases. However, there was no reliable correlation between these two indicators (p> 0.05).

**Table 2:** Sarcopenia rates at various BMI values in patients with pancreatic cancer.

		Sarcopenia		Total (n)
		Absent	Present	
BMI	Normal	12 (20.3%)	47 (79.7%)	59
	Increased	26 (50%)	26 (50%)	52
	Decreased	0	6 (100%)	6
Total		38 (33.5%)	79 (67.5%)	117

Comparative sex-specific indicators are shown in **Table 3**. We detected statistically significant SMI differences between males and females in the group of patients with pancreatic cancer (Group 1), with questionable differences in BMI values.

**Table 3:** Comparison of body composition by gender between two groups.

Group	Patients (n)	SMI cm^2^/m^2^		BMI kg/m^2^	
		men n = 56	women n = 61	men n = 56	women n = 61
1	117	46,8±7,8	37,4±5,9	24,5±5,1	24,9±5,2
2	60	57,6±3,9	43,1±3,2	23,6±4,2	24,8±5,3
		р = 0,000	р = 0,000	р>0,05	р>0,05

In the second group, the mean SMI value was 57.61±3.9 cm^2^/m^2^ for males and 43.14±3.2 cm^2^/m^2^ for females. Signs of CT-based sarcopenia with an evident weight loss (SMI 37.3 cm^2^/m^2^; BMI 15.9 kg/m^2^) were revealed only in one patient from this group—a 43-year-old man with chronic pancreatitis and alcohol abuse.

**Fig 3:**
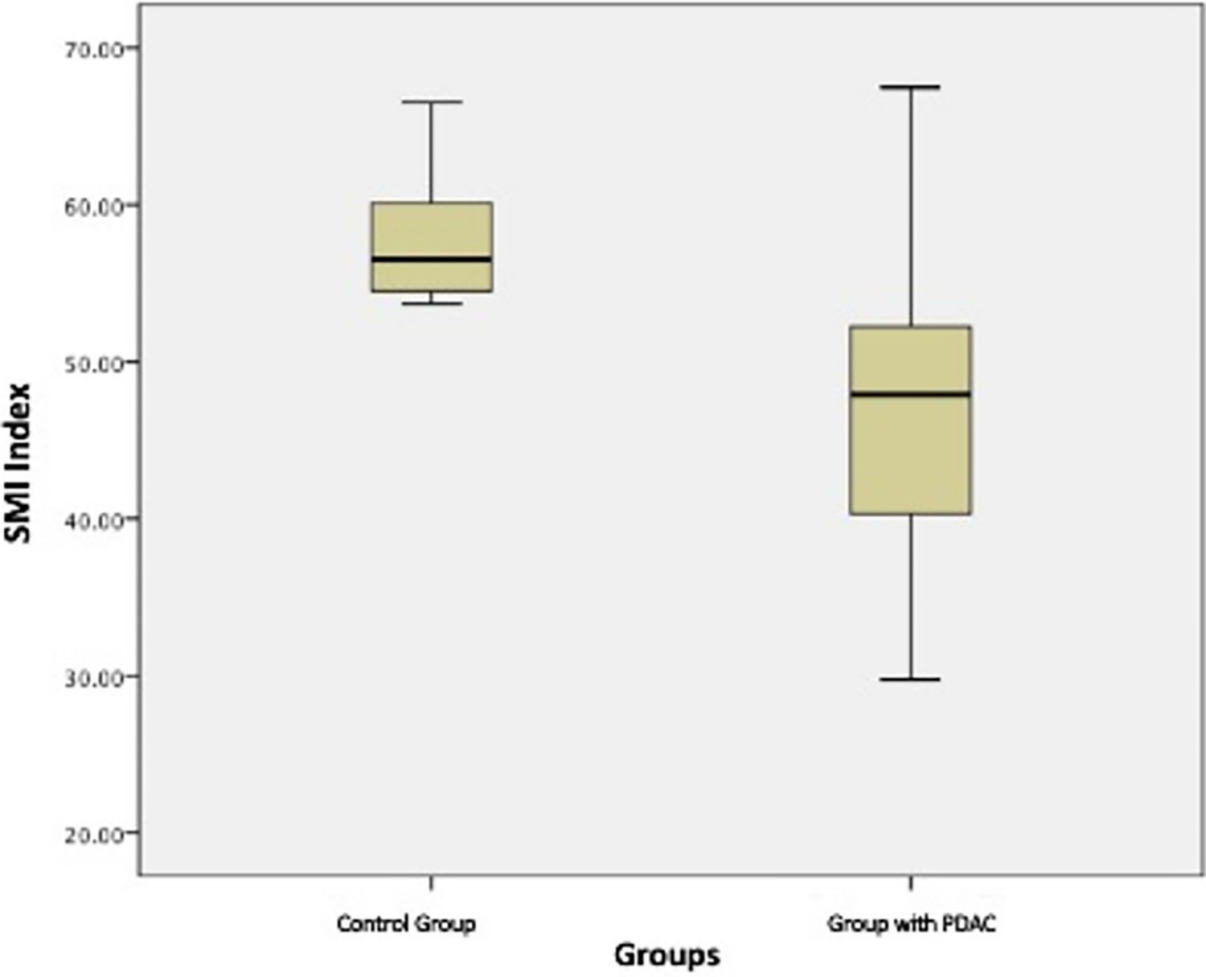
Comparative analysis of SMI in two groups.

**Fig 4:**
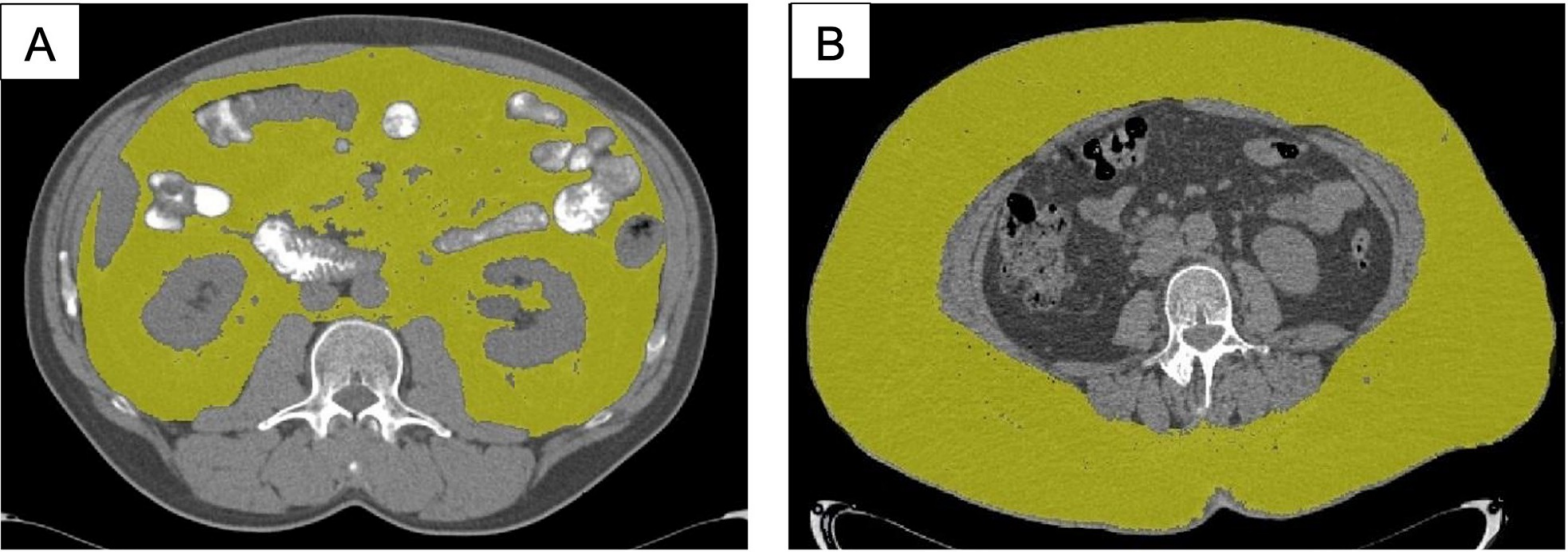
Axial segmented tomograms of two different patients with the same BMI, demonstration obesity of two different types with different distribution of fatty tissue: A—visceral obesity in men due to excess visceral fat tissue; B—subcutaneous obesity in women due to excess subcutaneous fat tissue.

**Fig 3** presents a comparative analysis of the skeletal-muscular index in the first and second group. The statistically significant difference between two groups was revealed for both males and females (p = 0.0001). A different distribution of fat in male and female patients was observed, however, without a statistically significant difference in BMI. **Fig 4** shows CT-studies in two different patients (male and female) with the same body mass index (BMI = 24.6 kg/m^2^), but with different types of fat distribution. A similar distribution of fatty tissue was observed in 64.3% of the males and 73.2% of females examined.

The results of the gender analysis, accounting for SMI, did not reveal a correlation of BMI with the body structure in patients with PDAC. At the same time, the types of fat distribution in men and women were different.

**Fig 5:**
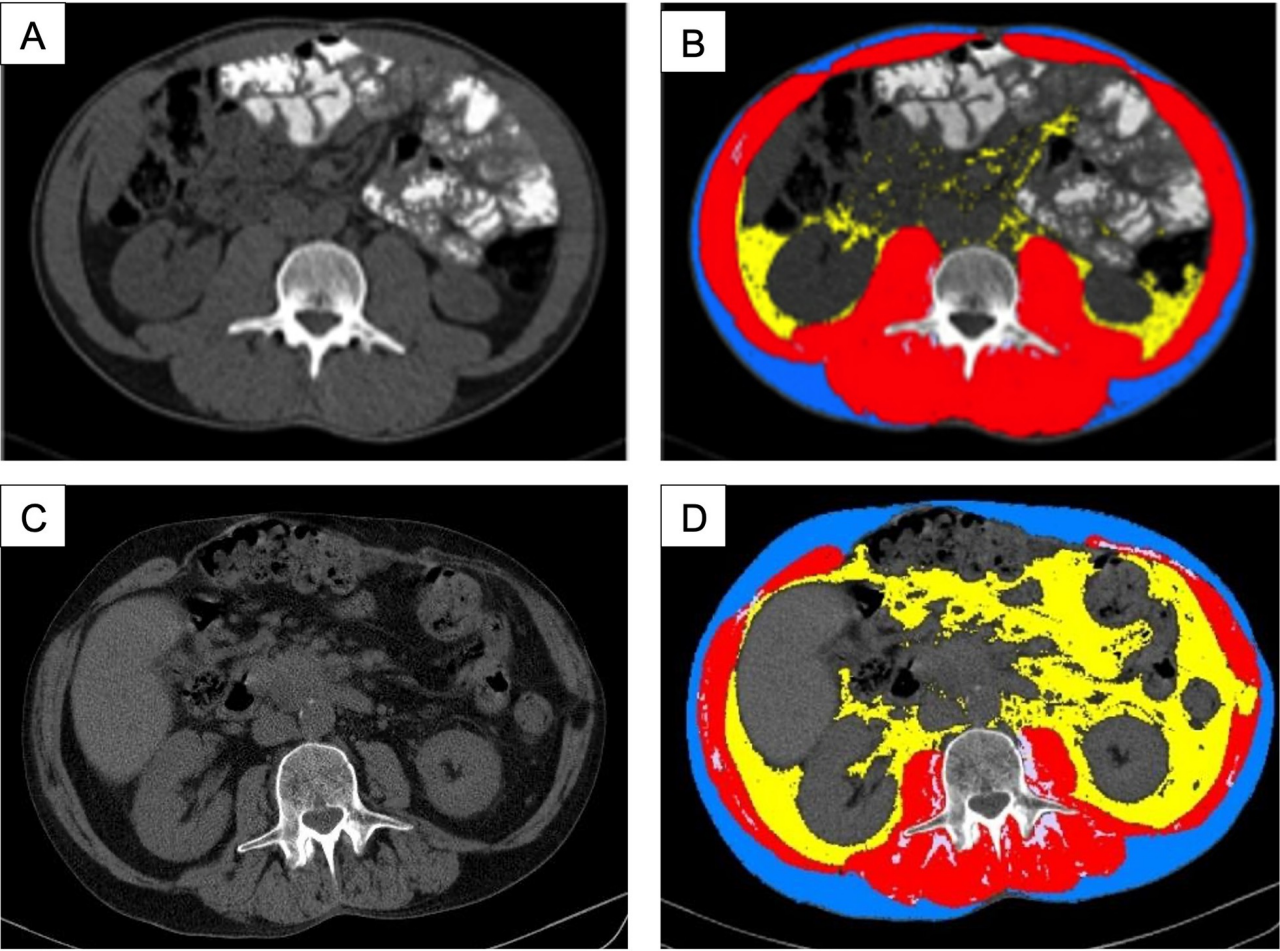
Axial CT-scans of two different patients with a healthy BMI value of 22.1 kg/m^2^. A and B: a patient without sarcopenia, both BMI and SMI are within the normal range, 22.1 kg/m^2^ and SMI 53.5 cm^2^/m^2^, respectively. A—initial CT-scan on the level of L3 vertebral body; B—segmented CT scan; the area of muscle tissue was 179.2 cm^2^; the total area of fatty tissue was 102.5.2 cm^2^.BA C and D: a patient with sarcopenia, BMI was normal with 22.1 kg/m^2^, SMI was decreased with 36.2 cm^2^/m^2^.DC A—initial CT image, performed at the L3 vertebral body level; B—segmented CT tomogram; the area of muscular tissue was 122.1 cm^2^; the total area of fatty tissue was 232.2 cm^2^.

**Fig 5** shows the axial slices of two different patients with a healthy BMI value (22.1 kg/m^2^). In the first patient, both BMI and SMI corresponded to normal values whereas in the second patient, the BMI also corresponded to normal values, but sarcopenia was observed. This is explained by the large area of adipose tissue and reduced muscle tissue area.

**Fig 6:**
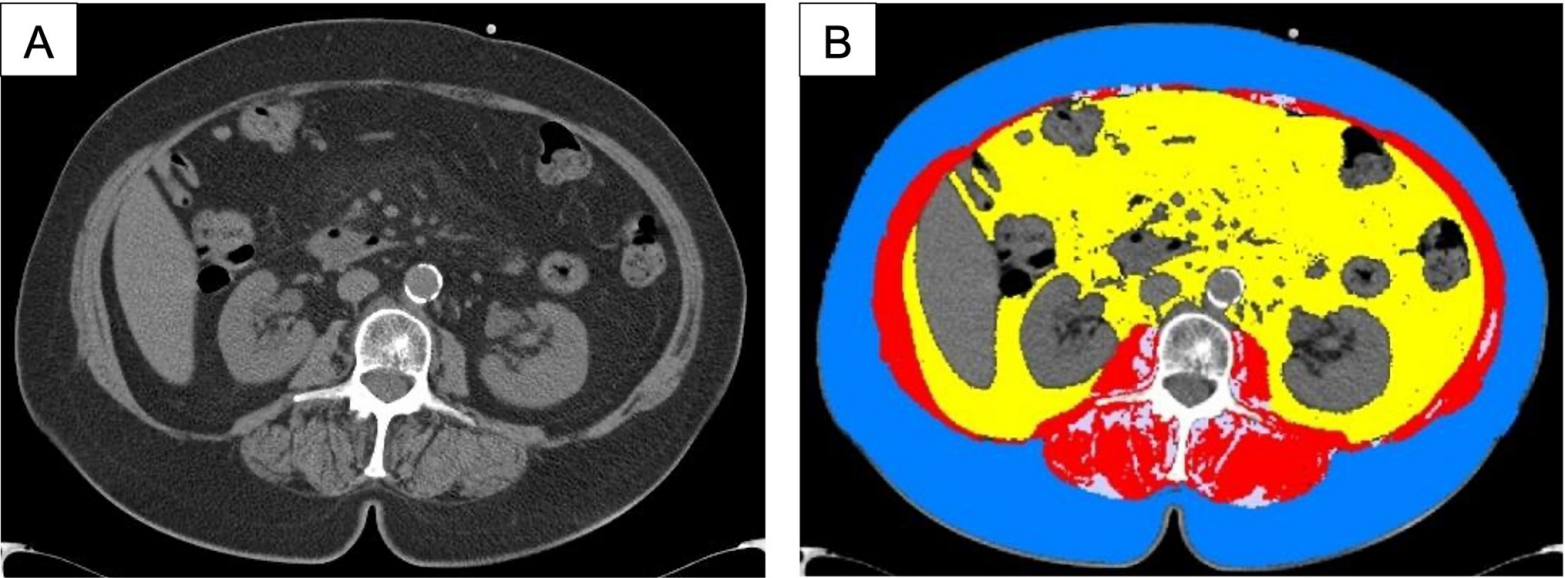
Axial CT-scans of patient with pancreatic ductal adenocarcinoma, performed at the L3 vertebral body level. BMI was increased with 27.7 kg/m^2^, SMI was decreased with 30.3 cm^2^/m^2^.DC A—initial CT image; B—segmented CT scan. The area of muscle tissue was 87.5 cm^2^, the total area of fat tissue—481.8 cm^2^.

As clinical example of “hidden” sarcopenia, we would like to present the following observation: male 49-year-old patient with pain in the epigastric area, weakness, loss of appetite, and weight loss (8 kg) within 2 previous months. A pancreatic ductal adenocarcinoma in pancreatic body was diagnosed and pathohistologically verified. Standard diagnostical CT protocol was extended with an assessment of BMI and SMI (**Fig 6**). By objectively excess weight (increased BMI: 27.7 kg/m^2^, normal values of 18.5 kg/m^2^ - 25 kg/m^2^), a significant reduction of SMI to 30.3 cm^2^/m^2^ (at normal values of >52.4 cm^2^/m^2^) was detected.

The average time spent by a radiologist on CT image processing to calculate the "skeletal-muscular index" was 10±3 minutes.

## Discussion

Sarcopenia was a very common condition in the first group of patients with pancreatic ductal adenocarcinoma (67.5%). Our results correspond to recent results of Chan W.Y. and Chok, who found sarcopenia in up to 65% of patients with PDAC and its association with poor survival outcomes after surgical treatment and chemotherapy [21]. In our group of PDAC patients, sarcopenia was present in patients with normal and with increased BMI. Thus, the results of our study allow us to consider BMI an inaccurate value for the assessment of body composition, as it does not reflect in the details of the human body structure.

Recently obtained evidence on the leading role of sarcopenia in the development of cachexia is of great clinical importance and allows a new look at many clinical problems. For example, today, the main criterion for the effectiveness of nutritional support is the increase in body weight. Meanwhile, in some cases, stabilization or increase in body weight may be due to an increase in the adipose tissue or edema with the background of increasing sarcopenia. Undoubtedly, the use of sectional imaging as CT and MRI allows a better treatment response assessment in cachexia.

Another important clinical aspect is the impact of sarcopenia on the toxicity of chemotherapy. Differences in toxicity can be attributed mainly to the heterogeneous composition of the patient’s body, which is vital for calculating the dosage of the chemotherapy drug. In this situation, the use of CT or MRI for the analysis of body composition seems to be reasonable since it does not require any additional examinations other than a priori used in the treatment of cancer patients.

Sarcopenic obesity is a clinical and functional condition which is defined by the coexistence of extended fat mass and sarcopenia. Nowadays, different definitions of sarcopenic obesity coexist and the diagnostic criteria and cut-offs are not established by consensus [22]. In our group of patients with pancreatic ductal adenocarcinoma, we reported sarcopenic obesity in patients with increased BMI and the presence of sarcopenia which was found in 56.4% of patients. Detection of this condition in patients with potentially resectable PDAC is important as it was shown to have higher morbidity and mortality risks and increased risk of developing such postoperative complication as postoperative pancreatic fistula [23, 24].

There is also impact of sarcopenic obesity on survival rate in patients undergoing chemotherapy to consider. In the study by Dalal et al. [25], patients with unresectable locally advanced PDAC were treated with bevacizumab in combination with capecitabine and radiation. The association of an increased loss in SMI of more than 3.8% and poorer survival rates were found (p = 0.02). In patients with sarcopenic obesity, the effect on survival rate was especially evident. According to the results of study by Kays et al. with retrospective design [26], 6 out of 53 patients with advanced PDAC, who received FOLFIRINOX had sarcopenic obesity. PDAC patients had a significantly shorter median overall survival compared with the other patients that were included in the study (10.4 months vs 16.1 months; p = 0.04). Similar results were shown in other groups of patients, for example in patients with lung and colon cancer. Prado et *al*. found [20] that sarcopenia was present in 15% of obese patients with lung and colon cancer. The mean survival in patients with sarcopenia was significantly lower than in patients without sarcopenia (1 and 21 months, respectively; p<0.001). In multifactor analysis, sarcopenic obesity had the most influence on the survival rate.

Sarcopenia has a clinical prevalence not only in chronic conditions and oncologic patients, but in acute conditions as well. Yoon et al. [27] reported a strong correlation of high visceral fat with low skeletal muscle volume with severity of acute pancreatitis. Visceral fat-to-muscle ratio had a stronger correlation with severity of acute pancreatitis than body weight or BMI. We also found BMI incapable of accurately assessing the body composition as well as the changes in body compositions within the time period completed.

One of the points of the study was the investigation of the length of time spent by a radiologist when calculating SMI. The average time spent by a radiologist on CT image processing to calculate the "skeletal-muscular index" was 10±3 minutes. At the same time, there was a tendency for this time to decrease from 10 to 5 minutes within one month as experience accumulated. Assessment of the patient’s body composition does not carry radiation exposure, as there is no need for additional scanning. Detection of sarcopenia in patients with PC requires only retrospective image analysis, which certainly increases the capabilities of the MDCT.

Recently, Paris MT et al. showed that it is possible to perform an automated body composition analysis using neural networks [28] where network segmentation took approximately 350 milliseconds/scan using modern computing hardware. The network showed excellent ability to analyze diverse body composition phenotypes and clinical cohorts which can create feasible opportunities to advance our capacity to predict health outcomes in clinical populations.

Since the SMI may be related to the clinical prognosis, it is advisable to report the presence or absence of sarcopenia and the changes in the patient’s body structure during prospective observation. The information about possible metabolic disorders reflected in the body structure is beneficial for physicians in the context of treatment planning.

The routine assessment of body composition with CT and MRI, and sarcopenia in particular, should be integrated in the diagnostic reports in the same way as densitometry in the diagnosis of osteoporosis.

## Conclusion

A differentiated approach to assessing body structure allows for a more accurate understanding of the clinical status of patients and the effectiveness of treatment. As skeletal muscle index may correlate with the prognosis, a radiologist should report decreased muscle mass (especially "hidden" sarcopenia or sarcopenic obesity) and the changes in the patients’ body structure. Even today this assessment in the daily clinical practice is far from being routine. Additionally, the use of CT will allow a better treatment response assessment in cachexia. The information about possible metabolic disorders reflected in the body structure is very useful for physicians in the context of treatment planning.

The most important aspect is the influence of sarcopenia on the toxicity of chemotherapy. Differences in toxicity can be explained mainly by the heterogeneous composition of the patients’ bodies, including patients with the same mass and body surface area. In this situation, the use of MDCT for the analysis of body composition is justified since it does not require any additional scanning as it is widely used in the primary diagnostic and treatment assessment of oncologic patients.

Assessment of sarcopenia and sarcopenic obesity should be integrated in clinical management of pancreatic ductal adenocarcinoma. All oncological patients that receive diagnostic CT studies can and should be screened for sarcopenia. However, until recently, this analysis required special software and medical personnel with sufficient skills. Today the emerging role of neural networks in assessment of body composition shows promising results and allows a cut in time of radiological assessment dramatically.

## Supporting information

S1 File (XLSX)

S2 File (XLSX)
